# Strontium and antimony serum levels in healthy individuals living in high‐ and low‐risk areas of esophageal cancer

**DOI:** 10.1002/jcla.23269

**Published:** 2020-04-22

**Authors:** Majid Mirzaee, Shahryar Semnani, GholamReza Roshandel, Mojgan Nejabat, Zahra Hesari, Hamidreza Joshaghani

**Affiliations:** ^1^ Golestan Research Center of Gastroenterology and Hepatology Golestan University of Medical Sciences Gorgan Iran; ^2^ Department of Biochemistry and Biophysics Faculty of Medicine Golestan University of Medical Sciences Gorgan Iran; ^3^ Department of Medicinal Chemistry School of Pharmacy Mashhad University of Medical Sciences Mashhad Iran; ^4^ Laboratory Sciences Research Center Golestan University of Medical Sciences Gorgan Iran; ^5^ Department of Laboratory Sciences Faculty of Paramedicine Golestan University of Medical Sciences Gorgan Iran

**Keywords:** antimony, esophageal cancer, high‐risk areas, low‐risk areas, strontium

## Abstract

**Background:**

It has been shown there is an upward trend for strontium (Sr) and antimony (Sb) levels from low‐risk (LR) to high‐risk (HR) areas of etiology of esophageal cancer in water, soil, and grains grown in Golestan province. In the present study, the serum levels of Sr and Sb were determined in healthy individuals living in these areas.

**Methods:**

This cross‐sectional study was performed on fasting blood serum of adult healthy individuals collected by cluster sampling. Subjects were divided into two groups, those living in either HR or LR areas. Strontium and antimony serum levels were measured using a graphite furnace atomic absorption spectroscopy.

**Results:**

A total of 200 volunteers were enrolled from which 96 persons (48%) and 104 persons (52%) were from either HR or LR areas, respectively. The sex distribution was 40.9% male and 59.1% female, and the average age of enrolled people was 50.9 years. The average strontium levels were 30.44 ± 4.05 and 30.29 ± 3.74 μg/L in LR and HR, respectively. It also has been shown the average antimony levels were 15.21 ± 3.40, 14.81 ± 3.17, 15.13 ± 3.62, and 15.07 ± 3.62 μg/L in LR, HR, urban, and rural populations, respectively.

**Conclusion:**

The serum levels of strontium and antimony were not significantly different in healthy adults living in high‐ and low‐risk areas of esophageal cancer. However, the average antimony serum levels in Golestan Province were above the reference interval in different countries.

## 1 INTRODUCTION

Golestan Province is located on one of the Asian esophageal cancer (EC) belts. The annual mortality rate of EC in this area is approximately 5800.^1^ Several studies have revealed the association of various factors, such as gene polymorphisms and inflammatory factors, with an increased risk of esophageal carcinoma.^2^, ^3^, ^4^, ^5^

East region of Golestan Province with a high incidence of EC is known as a high‐risk (HR) area, and the previous studies have indicated higher levels of strontium (Sr) and antimony (Sb) in water, soil, sediment, grains, and loess deposits. However, the levels of Sr and Sb in the west region of the province, which is considered as the low‐risk region, were lower. This suggests that Sr and Sb might have an impact on the incidence of EC.^6^, ^7^ Nevertheless, esophageal cancer has declined to less than half over the past thirty years, but there is an increasing trend of breast cancer in Golestan Province.^7^, ^8^

Strontium is the fifteenth abundant element on the earth that includes 0.02 to 0.03 percent of the earth's crust. Strontium is found in air, soil, and water and also is present in several pollutants due to human activities such as industry and agriculture.^9^ Strontium has a bone‐seeking property. It has been shown that some compounds containing strontium, such as strontium ranelate, stimulate osteoblasts to produce new bone as well as inhibit of the osteoclasts and finally prevent the reuptake of the bone.^10^ Some experimental studies have designed to investigate the anabolic mechanism of strontium in bone formation which may shed light into the carcinogenic properties of strontium.^11^, ^12^ The carcinogenic potential of strontium was strengthened considering its physiochemical properties which are similar to calcium and could increase ERK and activate RAS signaling pathways.^13^, ^14^

Antimony is an element with atomic number 51 that is located in row five and Group 15 of the periodic table. This element is the same group with elements such as arsenic and bismuth. Mainly in nature presents the trivalent Sb (III) and pentavalent Sb (IV) form of antimony.^15^ Exposure to antimony can occur via natural sources and industrial activities.^16^ Most toxic compounds of Sb are the antimony potassium tartrate that is in toxicity similar to arsenic oxide and caused alike diseases.^17^ Antimony containing compounds has been used successfully in the treatment of leishmaniasis for over half a century.^18^

The antiproliferative property of organo‐antimony against human breast and lung cancer cells is shown in the study of Polychronis and co‐authors. They also revealed that this compound act better than cisplatin.^19^ On the other hand, exposure to low dose of antimony has been shown to increase proliferation and migration of prostate cancer cells.^20^ The results of the study of Keshavarzi et al^6^, ^7^ showed the positive association between antimony and strontium with esophageal cancer incidence in Golestan Province.

In the present cross‐sectional study, we have evaluated strontium and antimony levels in the serum of individuals living in either HR or LR regions of Golestan Province. Previous studies have also shown that Sb and Sr levels in soil, grains, sediments, and loess deposits have exceeded the permissible limit in this region. This trespassed has been an increasing trend from the LR to the HR of the province.^6^, ^7^

Sb and Sr enter the human body via the soil‐plant‐food chain.^21^, ^22^, ^23^, ^24^ Hence, we hypothesized that by measuring the serum levels of these elements in people living in either high‐risk or low‐risk areas of esophageal cancer, we might find a correlation between higher serum levels of these elements and higher incidence of esophageal cancer.

## 2 MATERIALS AND METHODS

### 2.1 Population study

This study was descriptive, analytical, and cross‐sectional. Two hundred people were enrolled, 100 individuals from the HR area (kalale) and 100 people from the LR area (Kordkuy) of EC in Golestan Province. Healthy subjects and adult (over 30 years old) of both sexes were enrolled, and patients with cancer and blood hemolysis were omitted from the study.

Reagent: All reagents were analytical grade. Double‐distilled water (DDW) was used throughout the experiment. Other materials used in this study include concentrated hydrochloric acid, acetonitrile, concentrated nitric acid, ascorbic acid, Triton X‐100, Antifoam, and palladium chloride. The working solutions were prepared, including a diluent solution, modifier solution, and nitric acid 30% (for washing and soaking).

To build a diluent solution, we solved the amount of 1.0556 gr ascorbic acid in DDW and also 50 µL Triton X‐100 added into it, then were well mixed and after that added enough DDW to bring the volume up to 12.5 mL and finally, add to it 50 µL antifoams. To construct a palladium chloride solution, an amount of 10 mg of palladium chloride was added in 100 µL of concentrated hydrochloric acid, and then it was well mixed afterward bring the volume up to 10 ml with DDW.

### 2.2 Procedure

#### 2.2.1 Strontium measurement

**Figure 1:**
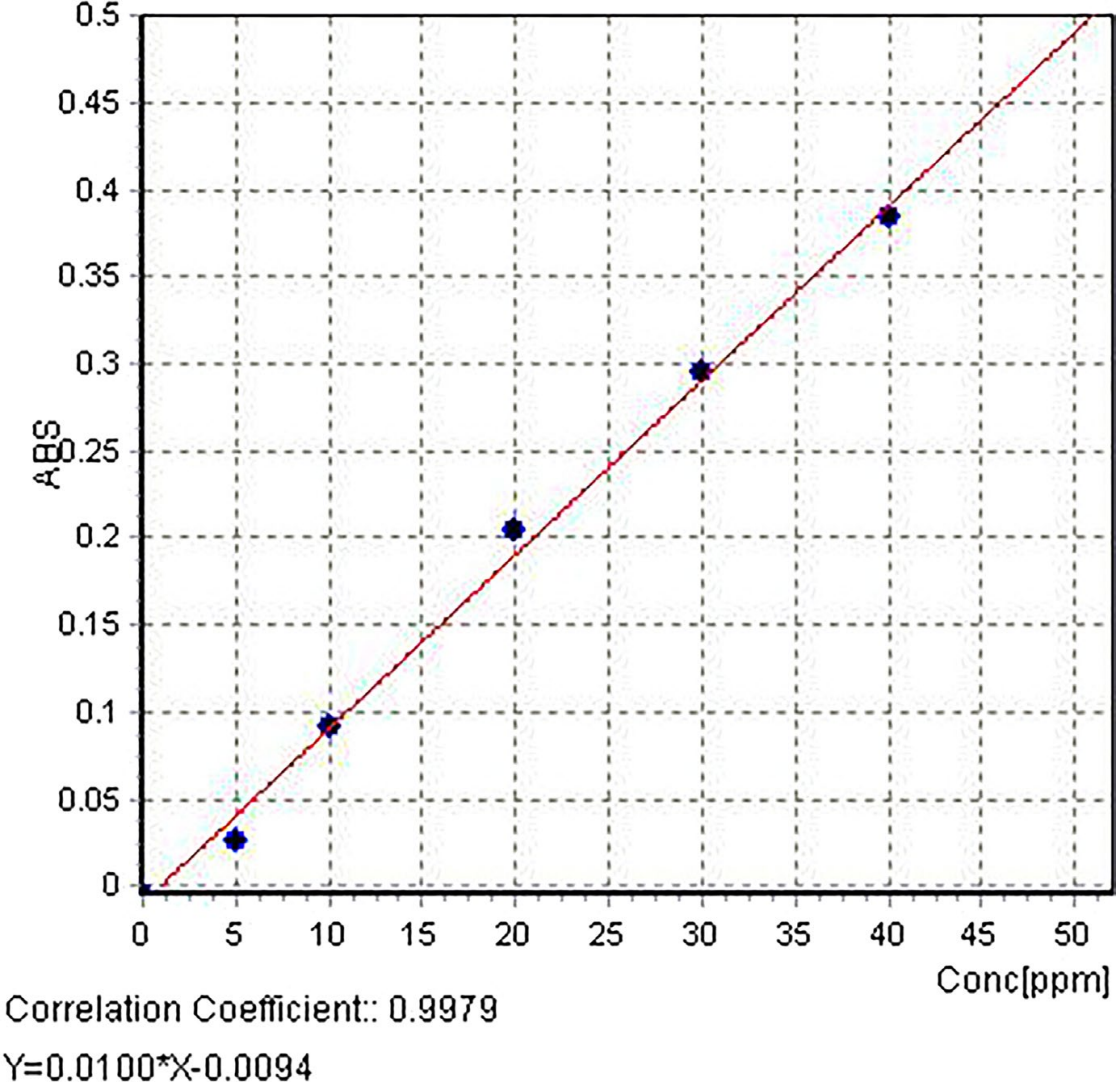
Strontium standard curve (The *x*‐axis and *y*‐axis represents the standard concentration and absorption, respectively)

Stock standard solution of strontium nitrate with a concentration of 1000 ppm was used. This solution was diluted, and series of working standard concentrations of 5, 10, 20, 30, and 40 μg/L were prepared. Furthermore, the standard solution without strontium nitrate was used as a calibration blank. A standard curve was constructed as shown in Figure 1.

To match the standard matrix with the serum sample matrix, we used acetonitrile for all dilutions. All the standard and frozen serum samples (after thawing) were mixed with acetonitrile at a ratio of 1:5. Acetonitrile deproteinizes the serum samples.

Atomic absorption spectrometer equipped with a graphite furnace (YOUNG LIN AAS 8010 model) with strontium Hollow Cathode Lamp was used to analyze the samples. The parameter requirements of the device for the determination of strontium are given in Table 1. For the analysis of test data, we used the Student *t *test analysis.

**Table 1:** GFAAS system parameters for measuring of strontium and antimony in serum

	Temp, °C	Ramp, s	Hold, s	Gas, mL/min (ON/OF)
Step				
Dry1 (Sr)	120	50	0	300 ON
Dry1 (Sb)	70	45	0	300 ON
Dry2 (Sr)	140	20	0	300 ON
Dry2 (Sb)	120	10	0	300 ON
Dry3 (Sb)	140	15	0	300 ON
Dry4 (Sb)	400	30	0	300 ON
Ash1 (Sr)	400	15	10	300 ON
Ash1 (Sb)	1000	10	5	300 ON
Ash2 (Sr)	400	0	3	OF
Ash2 (Sb)	1000	0	4	OF
Atom (Sr)	2700	0	3	OF
Atom (Sb)	2700	0	3	OF
Clean (Sr)	2800	0	3	300 ON
Clean (Sb)	2700	0	3	300 ON
Cool (Sr)	0	0	30	300 ON
Cool (Sb)	0	0	30	300 ON
Conditions				
Purge Gas (Sr and Sb)		Argon		
Mode (Sr and Sb)		Peak area		
Injection volume (Sr and Sb)		20 μL		
Wavelength (Sr)		460.7 nm		
Wavelength (Sb)		217.6 nm		
Furnace (Sr and Sb)		Pyrolytically coated graphite tube		
Accuracy and precision				
Recovery (Sr)		97%		
Recovery (Sb)		93%		
Coefficient of variation (Sr)		6%		
Coefficient of variation (Sb)		8%		
Standard deviation (Sr)		1.82		
Standard deviation (Sb)		0.94		
Limit of detection (Sr)		0.013		
Limit of detection (Sb)		0.001		

Abbreviations: GFAAS, Graphite furnace atomic absorption spectroscopy; Sb, antimony; Sr, strontium.

#### 2.2.2 Antimony measurement

**Figure 2:**
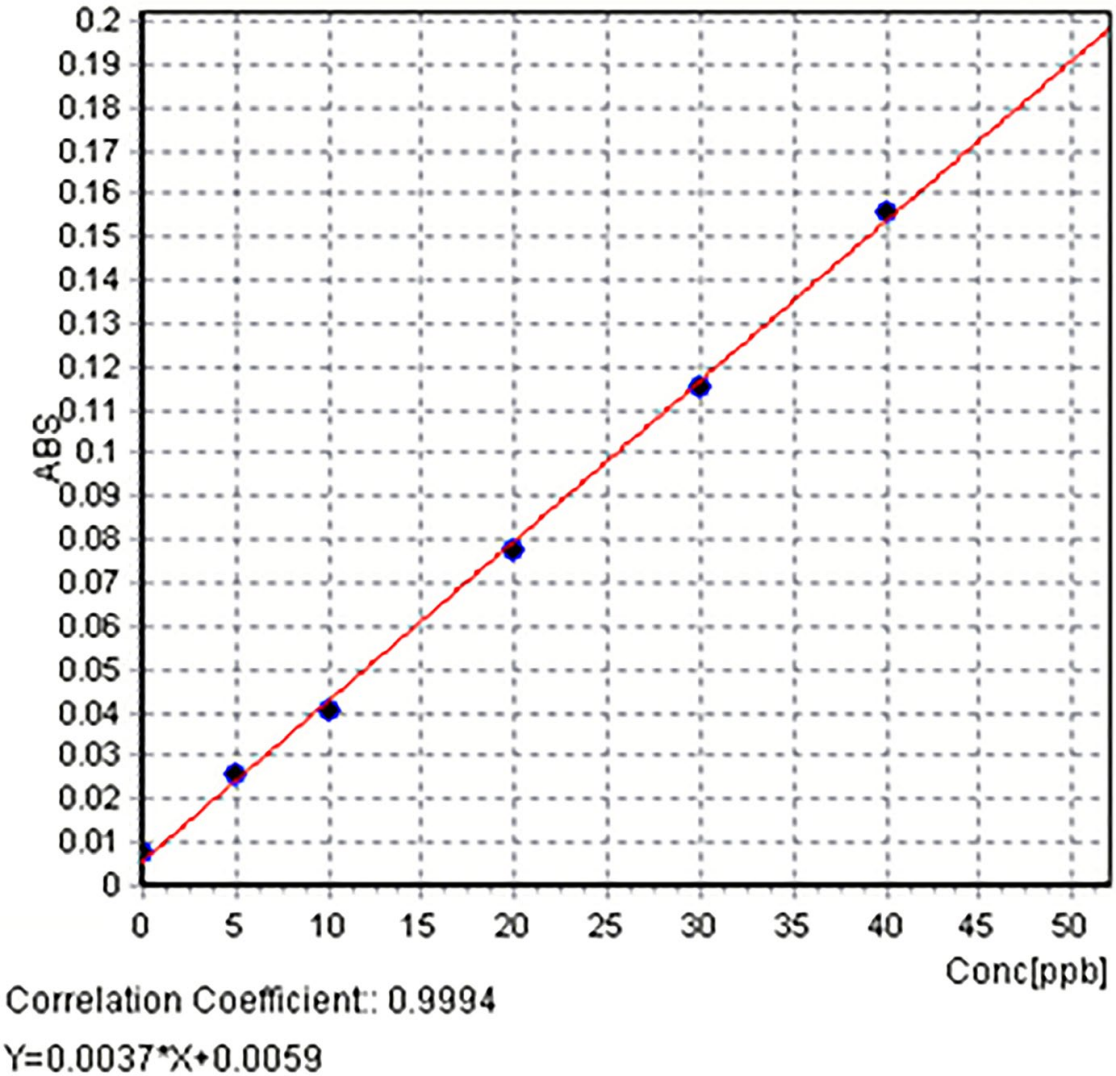
Antimony standard curve (The *x*‐axis and *y*‐axis represents the standard concentration and absorption, respectively)

A stock standard solution 1000 ppm from antimony chloride was used. This solution was diluted and made the series of working standard concentrations of 5, 10, 20, 30, and 40 μg/L as well as 0.0 ppm standard as a calibration blank. The standard curve is shown in Figure 2.

For evaluation of antimony, to match the standard matrix with serum sample matrix, nitric acid 10% for all dilutions. Each of the standard concentrations and also the frozen serum samples (after thawing) were mixed with diluent solution and a modifier solution with a volume ratio of 2:1:1.

Atomic absorption spectrometer equipped with a graphite furnace (YOUNG LIN AAS 8010 model) and without pieces auto‐sampler was used that which controls with workstation of AAS 8000 Atomic Absorption Spectroscopy software. Antimony hollow cathode lamp was used for reading samples. The parameter requirements of the device for the determination of antimony are given in Table 1. For the analysis of test data, we used *t* test analysis.

## 3 RESULTS

In the present study, a total of 200 people were enrolled in which 96 individuals (48%) from the HR area and 104 individuals (52%) from LR area also 40.9% men and 59.1% female. The mean age of participants was 50.9 with 14‐year standard deviation.

There was no significant difference between the serum concentrations of Sr in HR areas (30.29 ± 3.74 μg/L) compared with LR areas (30.44 ± 4.05 μg/L) (*P*‐value = .42) as well as in men (30.56 ± 3.69 μg/L) compared with women (30.24 ± 4.15 μg/L). There was no significant difference between the serum concentrations of strontium in urban population (30.01 ± 3.80 μg/L) compared with rural people (30.74 ± 4.10 μg/L). There was also no significant difference the serum concentrations of Sr in the age‐group 1 (30.92 ± 3.70 μg/L) compared with the age‐group 2 (29.87 ± 4.00 μg/L).

The results of this study also demonstrated no significant difference between the serum levels of Sb in HR areas (14.81 ± 3.17 μg/L) compared with LR areas (15.21 ± 3.40 μg/L) (*P*‐value = .42) and also the serum concentrations of that in men (15.59 ± 3.84 μg/L) compared with women (14.85 ± 3.04 μg/L). Comparison of serum Sb levels in urban population (15.13 ± 3.62 μg/L) did not show any significant difference compared with rural people (15.07 ± 3.62 μg/L). There was no significant difference between serum concentrations of Sb in the age‐group 1 (14.59 ± 3.14 μg/L) compared with the age‐group 2 (15.59 ± 3.55 μg/L).

To perform further analysis on the studied population, we evaluated West, East, and the whole province in terms of variables such as age, sex, and urban or rural residence, which results showed no significant differences in each of the groups (Table 2).

**Table 2:** Data analysis of strontium and antimony (μg/L)

Data analysis of strontium (μg/L)				Data analysis of antimony (μg/L)		
	Area			Area		
	West (LR)	East (HR)	*P*‐value	West (LR)	East (HR)	*P*‐value
WP	30.44 ± 4.05 (104/52%)	30.29 ± 3.74 (96/48%)	.42	15.21 ± 3.40 (104/52%)	14.81 ± 3.17 (96/48%)	.42

	Age‐group			Age‐group		
	Group (1) Below 52 y old	Group (2) Above 52 y old	*P*‐value	Group (1) Below 52 y old	Group (2) Above 52 y old	*P*‐value
West	31.29 ± 3.70 (39/41.5%)	29.48 ± 4.30 (55/58.5%)	.36	15.03 ± 3.45 (39/41.5%)	15.45 ± 3.58 (39/41.5%)	.36
East	30.62 ± 4.00 (47/54%)	30.40 ± 3.53 (40/46%)	.90	14.20 ± 2.80 (47/54%)	15.80 ± 3.55 (40/46%)	.90
WP	30.92 ± 3.86 (86/47.5%)	29.87 ± 4.00 (95/52.5%)	.48	14.59 ± 3.14 (86/47.5%)	15.59 ± 3.55 (95/52.5%)	.48

	Gender			Gender		
	Male	Female	*P*‐value	Male	Female	*P*‐value
West	30.64 ± 3.93 (41/43.6%)	29.92 ± 4.31 (54/62.1%)	.32	15.76 ± 4.01 (41/43.6%)	14.91 ± 3.10 (54/62.1%)	.29
East	30.46 ± 3.41 (33/37.9%)	30.56 ± 4.00 (54/62.1%)	.14	15.15 ± 3.66 (33/37.9%)	14.78 ± 2.99 (54/62.1%)	.30
WP	30.56 ± 3.69 (74/40.9%)	30.24 ± 4.15 (107/59.1%)	.88	15.59 ± 3.84 (74/40.9%)	14.85 ± 3.04 (107/59.1%)	.14

	Location			Location		
	Urban	Rural	*P*‐value	Urban	Rural	*P*‐value
West	29.99 ± 3.72 (59/62.8%)	30.63 ± 4.79 (35/37.2%)	.10	15.25 ± 3.38 (59/62.8%)	15.29 ± 3.82 (35/37.2%)	.24
East	30.03 ± 3.99 (32/36.8%)	30.81 ± 3.64 (55/63.2%)	.67	14.89 ± 2.84 (32/36.8%)	14.93 ± 3.52 (55/63.2%)	.42
WP	30.01 ± 3.80 (91/50.3%)	30.74 ± 4.10 (90/49.7%)	.46	15.13 ± 3.62 (91/50.3%)	15.07 ± 3.62 (90/49.7%)	.23

Note

Data are presented as mean ± SD (number of participants/percent of participants).

Abbreviations: HR, high‐risk areas; LR, low‐risk areas; WP, Whole of province.

## 4 DISCUSSION

Due to the potential of pentavalent antimony compounds for the treatment of Leishmaniasis, as well as indirect evidence for the carcinogenicity of strontium in drinking water, extensive studies have been conducted on the biological role of antimony and strontium.^7^, ^18^, ^25^, ^26^, ^27^, ^28^ Several studies found that trace elements such as antimony and strontium in drinking water are associated with cancer.^29^, ^30^, ^31^ Also, the anti‐cancer properties of some antimony compounds have been tested on the cell lines in vitro.^19^, ^20^, ^32^, ^33^ For these reasons, the importance of evaluating the serum levels of Sb and Sr in cancers, especially esophageal cancer, has been highlighted.

Keshavarzi et al^6^, ^7^ in a study with the aim of evaluation drinking water quality in the HR area for EC in Golestan Province indicated that villages in the HR areas of the province do not have a good‐quality drinking water and the levels of Sr and Sb have been exceeded the permissible limit in drinking water, soil, grain, loess deposits, and sediments of Golestan Province and showed the growing trend from LR to HR. Some studies have shown that Sr is present in the number of pollutants due to human activities (industry, agriculture, and transport) and enters into the body via a soil‐plant‐food chain.^21^, ^22^, ^23^, ^24^

On the other hand, other studies have revealed that the high urinary levels of these elements, which are due to prolonged exposure and make individuals susceptible to various diseases.^32^, ^34^, ^35^, ^36^, ^37^ Also, Makris et al^25^ in their study investigated the effects of various sources of drinking water upon urinary antimony concentration and eventually concluded that there is a significant relationship between using of the PET bottles (Polyethylene terephthalate) and urinary antimony concentrations.

Indeed, in all of these studies, we can see a relative increase of Sb and Sr in serum and urine, coordinated with an increase of them in the environment (water, soil, food, and drug). Even so, according to the results of the present study, there was no significant difference in serum levels of Sb and Sr between people who living in the East (area contains the high levels of Sb and Sr in soil and water) in comparison with those living in the West (area containing the low levels of Sb and Sr in soil and water). For industrial applications of Sb and Sr,^34^, ^38^ and because we can consider villages as industrial areas and cities as non‐industrial areas, we decided to survey differences of Sr and Sb concentrations in urban areas than rural areas. However, this study did not show any significant differences in serum levels of Sr and Sb between urban and rural residents. Perhaps the reason behind the lack of difference in serum Sr and Sb levels is that the serum and urinary concentrations of antimony and strontium in other studies were measured shortly after administration and exposure to them. So, the body does not have sufficient time to clear the serum and also excretion or absorption of antimony and strontium to the cells. While in the present study, we are faced with healthy subjects, who are continually over the years of his life were in contact with different levels of antimony and strontium. Therefore, maybe the body needs a lot of time for excretion of them into the urine and blood clearance. Sahilli et al^39^ in their study investigated the reaction between glutathione and potassium antimony tartrate and concluded that the 4‐hour exposure of Sb (III) to erythrocytes resulted in an increase in extracellular glutathione concentration and glutathione efflux. It is also concluded that about 98 percent of the total body content of strontium is implanted in bone tissue,^11^, ^12^ and almost 17 percent of strontium be excreted through the urine, which is the main way to dispose of strontium from the body.^34^

Since the risk of EC increases proportional with increasing of age,^2^ and older people are considered to have longer exposure to strontium and/or antimony, subjects in this study were divided into two age‐groups (above 52 years old and below 52 years old). Nevertheless, there was no significant difference between group 1 and group 2, while EC and the incidence of morbidity increase proportionally with age (such that in the seventh decade of life reaches its peak).^1^, ^2^

Bohanes et al^40^ demonstrated that gender is an independent prognostic factor for LEC (Localized Esophageal Cancer) and MEC (Metastatic Esophageal Cancer). Moreover, the incidence of esophageal cancer has shown a marked increase in men compared with women.^2^ In addition, Nriagu and Callum^41^, ^42^ linked antimony exposure to man occupational activities.

It have been shown that the calcium serum level was significantly associated with the risk of breast cancer in women.^43^, ^44^, ^45^, ^46^, ^47^ Strontium may react with estrogen and to induce carcinogenesis in young women.^35^, ^48^ Other studies have demonstrated the breast cancer cells, such as MCF‐7, expressed a cell‐surface receptor called "calcium‐sensing" (CaR) which can be activated with Sr^49^ CaR mediates the expression of estrogen receptor (ER), which is activated by calcium.^50^ Chen has been inferred that strontium similar to calcium can activate ER through the CaR, and its effects are alike to estrogen.^35^ Since there is an increasing trend of breast cancer in Golestan,^7^, ^8^ therefore, we assumed might be different levels of Sr and Sb in the serum of men and women. Contrary to our assumption, the difference between men and women in West, East, and the whole of the province was not significant. This could indicate that probably occupational contamination has no significant role in increasing the serum levels of Sb and Sr.

On the other hand, it is important to note that in studies conducted by Forlanini, Heitland and Bocca^51^, ^52^, ^53^ with the aim of determining a reference interval for antimony were reported the levels of 0.1‐1.48, 0.1‐1.3, and 0.20‐0.57 μg/L, respectively. While the results of our study demonstrate that the range of antimony concentration in Golestan Province is between 9 and 26 μg/L. With irrespective the lack of difference between HR and LR, if we look at the numbers obtained and compare it with the reference interval in other countries, can see that we are dealing with higher numbers.

The study performed by Semnani et al^54^ in Golestan Province showed that EC in this region has been declined in more than half during the last past 30 years, whereas items of breast and colorectal cancer are increased. Regarding the results of the present study, increased Sb and Sr levels may have an inhibitory effect on esophageal cancer and an induction effect on breast and colorectal cancer in this region.

## 5 CONCLUSIONS

The serum levels of strontium and antimony were not significantly different in healthy adults living in high‐ and low‐risk areas of esophageal cancer. However, the average antimony serum levels in Golestan Province were above the reference interval in different countries.
